# Development of the Theta Comparative Cell Scoring Method to Quantify Diverse Phenotypic Responses Between Distinct Cell Types

**DOI:** 10.1089/adt.2016.730

**Published:** 2016-09-01

**Authors:** Scott J. Warchal, John C. Dawson, Neil O. Carragher

**Affiliations:** Institute of Genetics and Molecular Medicine, Cancer Research UK Edinburgh Centre, University of Edinburgh, Edinburgh, United Kingdom.

## Abstract

*In this article, we have developed novel data visualization tools and a Theta comparative cell scoring (TCCS) method, which supports high-throughput* in vitro *pharmacogenomic studies across diverse cellular phenotypes measured by multiparametric high-content analysis. The TCCS method provides a univariate descriptor of divergent compound-induced phenotypic responses between distinct cell types, which can be used for correlation with genetic, epigenetic, and proteomic datasets to support the identification of biomarkers and further elucidate drug mechanism-of-action. Application of these methods to compound profiling across high-content assays incorporating well-characterized cells representing known molecular subtypes of disease supports the development of personalized healthcare strategies without prior knowledge of a drug target. We present proof-of-principle data quantifying distinct phenotypic response between eight breast cancer cells representing four disease subclasses. Application of the TCCS method together with new advances in next-generation sequencing, induced pluripotent stem cell technology, gene editing, and high-content phenotypic screening are well placed to advance the identification of predictive biomarkers and personalized medicine approaches across a broader range of disease types and therapeutic classes.*

## Introduction

The treatment of complex disease in human populations is often confounded by the broad heterogeneity in the mechanisms responsible for the generation and evolution of disease-affected cells. Within an individual patient and between genetically distinct patients, such heterogeneity in disease mechanisms contributes to poor drug responses and relapses observed in the clinic.^1,2^ Sequencing of the human genome and advances in characterizing patient disease at genetic and proteomic levels support the personalized medicine concept of treating each individual patient with the most appropriate therapy for their disease.^3,4^

Key to the personalized medicine approach is the identification of biomarkers, which can be readily measured in patient samples to predict drug response. Many such biomarkers, for example, BRAF V600E (Melanoma/Colorectal Cancer); EGFR (Nonsmall cell lung carcinoma); and HER-2 (Breast Cancer), are associated with monitoring activation state and mutation status of known drug targets to predict response to therapy.^5–7^ Thus, the personalized medicine approach is well suited to target-directed drug discovery strategies where target pathways are clearly defined. However, such target-directed personalized medicine strategies are unsuitable for many complex diseases and drugs discovered by phenotypic drug discovery, where they are not defined by a single target or the mechanism-of-action and therapeutic targets remain to be fully elucidated.^8,9^ Thus, more unbiased approaches to the identification of biomarkers, including genetic or pathway signatures, which predict drug response are required to expand the personalized medicine concept across complex disease types and therapeutic classes.

Comparative analysis of well-characterized panels of human cell lines derived from distinct individuals has many applications in basic research, drug discovery, and personalized medicine. Genomic and transcriptional profiling of cancer cell line panels, such as the National Cancer Institute 60 human tumor cell line drug screen collection, provide a genetic context to comparison of cell function and drug sensitivity, supporting biomarker discovery and drug mechanism-of-action analysis.^10^

High-throughput *in vitro* pharmacogenomic studies across larger cancer cell line panels have been established and provide valuable resources, such as the Cancer Cell Line Encyclopedia (CCLE) from the Broad Institute www.broadinstitute.org/ccle/home and the Catalogue of Somatic Mutation in Cancer Cell Lines project from the Sanger Institute http://cancer.sanger.ac.uk/cell_lines, which facilitate pharmacogenomic analysis. Such drug sensitivity profiling across genetically defined cell panels is now routinely implemented in academia and industry to identify biomarkers of response to support disease positioning and patient stratification strategies, or to further understand drug mechanism-of-action at genetic and proteomic levels.^11,12^

To our knowledge, all examples of *in vitro* high-throughput pharmacogenomic studies carried out to date utilize either a concentration of a drug that gives half-maximal response (EC_50_) or concentration of a drug that gives half-maximal inhibition of cell proliferation (GI_50_) value obtained by standard cell viability assays as the primary phenotypic endpoint for correlating drug sensitivity with genomic or transcriptomic datasets. While the GI_50_ and EC_50_ measurements of cell viability provide the necessary univariate value for quantifying drug sensitivity across a panel of cell lines, this method has several limitations.

Accurate measurement of EC_50_ or GI_50_ values is dependent upon obtaining full sigmoidal dose–response curves for each drug or compound tested in the assay. Dose–response curves and thus the EC_50_/GI_50_ calculations are prone to fluctuation dependent upon assay conditions, including cell culture media, atmospheric conditions, cell line health and cell line batch variation, and the type of viability assay reagents used. Indeed, comparative analysis of large pharmacogenomic studies published by the Broad and Sanger institutes have resulted in reports of inconsistency between the genetic signatures of drug sensitivity assigned to drugs shared between both studies.^13,14^ Cell viability assays and EC_50_/GI_50_ values are also not suitable for the majority of disease models, which are not defined by a single viability endpoint, or for quantifying drug response in more complex and physiologically relevant cell assays such as three-dimensional (3D) coculture models.

High-content imaging enables the quantification of multiple phenotypic cellular endpoints with high spatial and temporal resolution supporting drug sensitivity testing across more complex *in vitro* assays including 3D and coculture models.^15^ Image-based phenotypic profiling combined with multiparametric analysis methods allows detailed characterization of drug mechanism-of-action and classification of phenotypic response, including identification of novel compound target associations based upon similarity of multiparametric phenotypic fingerprints with annotated reference compound sets.^16–22^

The application of multiparametric biological profiling of compound libraries, by image-informatics and biospectra analysis methods, supports unbiased approaches to mechanism-of-action classification and identification of structure–activity relationships independent of target hypothesis.^23–25^ While multiparametric methods incorporating machine learning and artificial neural networks have steadily evolved to support phenotypic profiling across several cell types,^18,20,26^ there are few studies that perform comparative multiparametric phenotypic analysis between distinct cell types in drug discovery. Thus, despite over 15 years of continued development in the high-content screening field, there are few reports of pharmacogenomic studies performed across the diversity of complex phenotypes that can be measured by multiparametric high-content analysis approaches. A number of challenges that must be overcome to apply high-content phenotypic profiling to pharmacogenomic or pharmacoproteomic strategies include the following: defining relevant phenotypic endpoints, which appropriately quantify drug sensitivity; quantifying diverse phenotypic response across a dose response; visualizing multiple diverse phenotypes elicited across dose response and distinct cell panels; and reducing multiparametric high-content analysis of cell phenotype to a robust univariate metric for correlating drug sensitivity with genomic or proteomic datasets.

The goals of this study were to develop a robust and scalable method for quantifying diverse multiparametric high-content phenotypes and distinct compound-induced phenotypic response across a panel of cell lines. We describe the optimization of a high-content cell-painting assay to enable analysis of a broad range of cell phenotypes across a panel of clinically relevant breast cancer subtypes. We present new methods for normalizing and displaying distinct and dose-dependent multiparametric high-content phenotypic response across multiple cell types. We introduce the development and application of the “Theta Comparative Cell Scoring” (TCCS) method for calculating distinct phenotypic response between cell types. We describe the broad utility of the TCCS method in providing a univariate metric for quantifying distinct phenotypic response between compounds tested in the same cell and for compounds tested across multiple cell types. We make available the source code to enable application of TCCS across large high-content datasets. We present proof-of-principle data from a small compound screen performed on a panel of eight breast cancer cells representing four well-characterized and clinically relevant subtypes. We demonstrate the ability of our TCCS method to cluster cell types, which have similar or distinct phenotypic response to individual compounds, to guide patient stratification hypothesis and facilitate pharmacogenomic or proteomic studies. We discuss the potential impact of this approach upon extending the application of *in vitro* pharmacogenomic and personalized medicine strategies across a wider range of disease areas and therapeutic classes.

## Materials and Methods

### Cell Culture

Eight breast cancer cell lines were selected for their stratification of four well-characterized breast cancer clinical subtypes (*Table 1*). Authenticated cell lines were acquired from the American Type Culture Collection and carefully monitored for morphological changes to ensure authenticity. Cell lines were cultured in either Dulbecco's Modified Eagle's Medium (HCC1954, MCF7, KPL4, MDA-MB-231, MDA-MB-157, and SKBR3) or Roswell Park Memorial Institute-1640 (HCC1569 and T47D) supplemented with 10% fetal bovine serum and 2 mM l-glutamine and incubated at 37°C, 5% CO_2_. Two thousand five hundred cells were seeded into each of the inner 60 wells of 96-well plates (#165305; Thermo) in 100 μL media and incubated for 24 h before compound treatment. Outer wells of plates were filled with 100 μL phosphate-buffered saline (PBS).

**Table 1.** Panel of Breast Cancer Cell Lines

		Mutation Status	
Cell Line	Subclass	PTEN^a^	PI3K^b^
MCF7	ER^c^	WT^d^	E545K
T47D	ER	WT	H1047R
MDA-MB-231	TN^e^	WT	WT
MDA-MB-157	TN	WT	WT
HCC1569	HER2^f^	WT	WT
SKBR3	HER2	WT	WT
HCC1954	HER2	?^g^	H1047R
KPL4	HER2	?	H1047R

^a^ Phosphatase and tensin homolog.

^b^ Phosphoinsitide-3-kinase.

^c^ Estrogen receptor.

^d^ Wild type.

^e^ Triple negative.

^f^ Human epidermal growth factor receptor 2.

^g^ Lack of consensus regarding the mutational status of those cell lines.

ER, estrogen receptor; HER2, human epidermal growth factor; PI3K, phosphoinositide 3-kinase; PTEN, phosphatase and tensin homolog; TN, triple negative; WT, wild type.

### Compound Treatment

A panel of well-annotated compounds purchased from commercial suppliers (*Table 2*) were prepared as 10 mM stock solutions in dimethyl sulfoxide (DMSO). 1,000× compound plates were then created with semi-log dilutions in DMSO. Each plate contained six wells of 0.1% DMSO as a negative control and six wells of 200 nM staurosporine as a positive control. Following compound addition, cell assay plates were incubated at 37°C, in 5% CO_2_ incubator for an additional 48 h before fixation, staining, and high-content imaging.

**Table 2.** Compounds

Compound	Class	Sub-Class	Supplier	Cat. No.
Paclitaxel	Microtubule disrupting	Microtubule stabilizer	Sigma	T7402
Epothilone B	Microtubule disrupting	Microtubule stabilizer	Selleckchem	S1364
Colchicine	Microtubule disrupting	Microtubule destabilizer	Sigma	C9754
Nocodazole	Microtubule disrupting	Microtubule destabilizer	Sigma	M1404
Monastrol	Microtubule disrupting	Eg5 kinesin inhibitor	Sigma	M8515
ARQ621	Microtubule disrupting	Eg5 kinesin inhibitor	Selleckchem	S7355
Barasertib	Microtubule disrupting	Aurora B inhibitor	Selleckchem	S1147
ZM447439	Microtubule disrupting	Aurora B inhibitor	Selleckchem	S1103
Cytochalasin D	Actin disrupting	Actin disrupter	Sigma	C8273
Cytochalasin B	Actin disrupting	Actin disrupter	Sigma	C6762
Jasplakinolide	Actin disrupting	Actin stabilizer	Tocris	2792
Latrunculin B	Actin disrupting	Actin stabilizer	Sigma	L5288
MG132	Protein degradation	Proteosome	Selleckchem	S2619
Lactacystin	Protein degradation	Proteosome	Tocris	2267
ALLN	Protein degradation	Cysteine/calpain	Sigma	A6165
ALLM	Protein degradation	Cysteine/calpain	Sigma	A6060
Emetine	Protein synthesis	Protein synthesis	Sigma	E2375
Cycloheximide	Protein synthesis	Protein synthesis	Sigma	1810
Dasatinib	Kinase inhibitor	Src-EMT	Selleckchem	S1021
Saracatinib	Kinase inhibitor	Src-EMT	Selleckchem	S1006
Lovastatin	Statin	Statin	Sigma	PHR1285
Simvastatin	Statin	Statin	Sigma	PHR1438
Camptothecin	DNA damaging agent	Topoisomerase 1 inhibitor	Selleckchem	S1288
SN38	DNA damaging agent	Topoisomerase 1 inhibitor	Selleckchem	S4908

Src-EMT, Src kinase and Epithelial-Mesenchymal Transition inhibitor.

### Imaging

We adapted the cell painting protocol from Gustafsdottir *et al.*^27^ to optimize the cell staining across the eight selected breast cancer cell lines. Specific modifications to the original protocol by Gustafsdottir *et al.*^27^ were implemented to circumvent morphological changes induced upon the MDA-MB-231 cell line, which was particularly sensitive to live cell staining. The modifications included using all stains on postfixed samples and adjusting concentrations of reagents to optimize staining across the cell lines. The following adapted cell painting protocol was therefore applied to our breast cancer cell panel.

After a 48-h incubation in the presence of compounds, an equal volume of 8% paraformaldehyde (PFA) was added to the culture media of each well resulting in a final concentration of 4% PFA fixation buffer; the plates were then incubated at room temperature for 20 min, followed by three washes in 100 μL PBS. Permeabilization was performed with the addition of 50 μL 0.1% Triton-X100 to each well and incubation at room temperature for 20 min followed by three washes in 100 μL PBS.

The staining solution was prepared in a blocking buffer consisting of 1% bovine serum albumin in PBS (*Table 3*). Thirty microliters of staining solution was added to each well and incubated in darkness at room temperature for 30 min followed by three washes in 100 μL PBS, with no final aspiration. Plates were then sealed (#PCR-SQ plate max) and imaged immediately.

**Table 3.** Stains and Concentrations Used in the Modified Cell-Painting Protocol

Stain	Structure Labeled	Wavelength (ex/em [nm])	Concentration	Cat. No.; Supplier
Hoechst 33342	Nuclei	387/447	2 μg/mL	#H1399; Mol. Probes
SYTO14	Nucleoli	531/593	3 μM	#S7576; Invitrogen
Phalloidin 594	F-actin	562/624	0.85 U/mL	#A12381; Invitrogen
Wheat germ agglutinin 594	Golgi and plasma membrane	562/624	8 μg/mL	#W11262; Invitrogen
Concanavalin A 488	Endoplasmic reticulum	462/520	11 μg/mL	#C11252; Invitrogen
MitoTracker DeepRed	Mitochondria	628/692	600 nM	#M22426; Invitrogen

Plates were imaged on a Molecular Devices ImageXpress^®^ Micro XLS, six fields of view were captured per well using a 20× objective and five filters, DAPI (387/447 nm), FITC (482/536 nm), Cy3 (531/593 nm), TxRed (562/642 nm), and Cy5 (628/692 nm). Exposure, binning, and other image settings were not altered between cell lines.

### Image Analysis

Images were analyzed using CellProfiler v2.1.1^19^ to extract 309 features (*Supplementary Table S1*; Supplementary Data are available online at www.liebertpub.com/adt). Briefly, cell nuclei were segmented from the nuclei image based on intensity and shape, and used as seeds to segment cell areas in the other channels. Subcellular structures such as nucleoli and endoplasmic reticulum speckles were segmented and assigned to parent objects. From these objects, measurements such as size, shape, and spatial distribution were measured. The final CellProfiler settings applied in this study were created by iteratively adjusting the parameters and assessing the performance of cell segmentation by eye across multiple drug treatments for all cell types under evaluation, to ensure the most robust segmentation for each distinct cell type, and drug-induced phenotype is achieved.

### Data Preprocessing

Out of focus and low-quality images were detected and removed by filtering on saturation and focus measurements provided in the CellProfiler output. Image averages of single object measurements from CellProfiler were aggregated by taking the median of each measured feature per image. Features were normalized and standardized on a plate-by-plate basis by dividing each feature by the median DMSO response for that feature and then scaled by a *z*-score to have a mean of 0 and a standard deviation of 1. Feature selection was performed by calculating pair-wise correlations of features and removing one of a pair of features that have correlation greater than 0.9 and removing features with very low or zero variance, using the findCorrelation and nearZeroVar functions in the caret R package.^28^

### Quantifying Differential Morphological Responses by TCCS

Principal component analysis (PCA) was performed and the data were then centralized to the DMSO centroid. This was carried out by calculating the mean of principal component (PC) 1 and 2 for the DMSO subset of the data, and then subtracting this from the PC values. With each data point as a vector in two-dimensional (2D) space formed by the first two PCs, the norm of each vector was calculated, returning a Euclidean distance of each data point from the DMSO centroid. Then, the angles between each vector and a reference vector (0, 1) were calculated and denoted as theta (θ). The reference vector is arbitrarily set as a vector along the *x*-axis and enables easy comparison between the polar coordinate histograms of the PCA biplot in Cartesian coordinates. For replicates, median values of PCs were calculated before calculating vectors; this simple approach avoids the pitfalls in calculating the mean of circular quantities—for example the arithmetic mean of 1° and 359° is 180°, despite the close proximity of the values in polar coordinates.

As any perturbations that do not produce morphological changes will be indistinguishable from negative control values, these points were found clustered within the negative control cloud in a scatter-plot of the first two PCs. As these compounds are centered on the origin (0, 0), the angles calculated from their vectors are uniformly distributed in all directions and meaningless as a phenotypic direction. Therefore, a minimum distance from the DMSO centroid was determined as 1 standard deviation of the vector distances from the origin, and compounds within this distance were defined as inactive in our assay and not used in further calculations. Active compounds were only included if they fell beyond this minimum limit for all the eight cell lines.

To calculate the phenotypic difference between compounds tested within the same cell line or a compound tested across different cell lines using the vector analysis described above, the absolute difference between the two theta values can be used. However, as any difference greater than 180° and approaching 360° starts to reflect morphologies becoming more similar, the absolute difference values have to be constrained between 0° and 180°; this is carried out for values greater than 180 by subtracting the value from 360, for example, 190° will become 170°. We named the method “Theta-Comparative–Cell-Scoring” to reflect the use of vectors applied to multiparametric high-content data to quantify distinct phenotypic response between cell types.

### Data and Code Availability

The CellProfiler pipelines, numeric data, and R code to run the analyses and generate the figures are available at github.com/swarchal/TCCS_paper

## Results

### High-Content Phenotypic Comparisons Between Morphologically Distinct Breast Cancer Cell Subtypes

We have modified the cell painting assay previously applied to the osteosarcoma cell line U2OS cells^27^ to a panel of breast cancer cell lines representing clinically relevant subtypes. Eight breast cancer cell lines representing four pairs for each of the following clinical subtypes: estrogen receptor (ER)-positive, triple negative, human epidermal growth factor receptor 2 (Her2)-postive/Phosphatase and tensin homolog (PTEN) and phosphoinositide 3-kinase (PI3K) wild type, and Her2-positive/PTEN and PI3Kmut were selected for this study (*Table 1*).

**Fig. 1.**
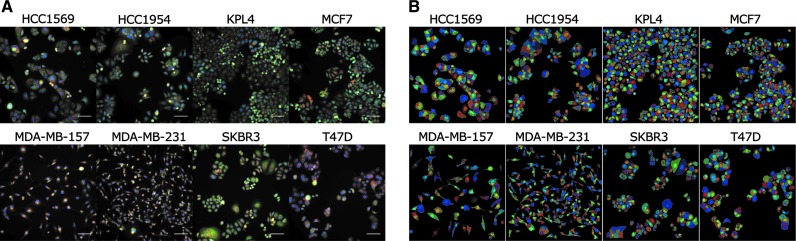
Cell painting assay applied to eight distinct breast cancer cell lines. **(A)** Composite image of cell lines treated with 0.1% DMSO. Channels used: *Red*—MitoTracker DeepRed (mitochondria); *Green*—Concanavalin A (endoplasmic reticulum); *Blue*—Hoechst33342 (nuclei). Scale bars: 100 μm. **(B)** Image masks from CellProfiler showing nuclei and cell body segmentation. DMSO, dimethyl sulfoxide.

The modified cell painting assay was optimized to enable the CellProfiler image analysis software to segment individual cells for each well and extract features, which provide detailed morphological analysis of individual breast cancer cell phenotypes. Representative images of the eight breast cancer cells stained with the modified cell-painting protocol are displayed in three channels in *Figure 1A* and respective cell segmentation masks generated by CellProfiler analysis are shown in *Figure 1B*. As the breast cancer cell lines look inherently different from one another (*Fig. 1*), detecting differential phenotypic changes between them requires normalization against the negative control phenotype for each cell line. This was performed by dividing each feature by the median DMSO value for that feature on a plate-by-plate basis followed by *z*-scoring each feature individually for all cell lines. Normalization in this manner achieved two objectives: (1) removing any batch effects that may be present across plates and (2) normalizing all phenotypic measurements as standardized fold changes from the negative control values per cell line. PCA was then performed on the normalized dataset of all cell lines using the prcomp function in R.

### Quantifying Differential Morphological Response Between Cell Lines to the Same Compound

**Fig. 2.**
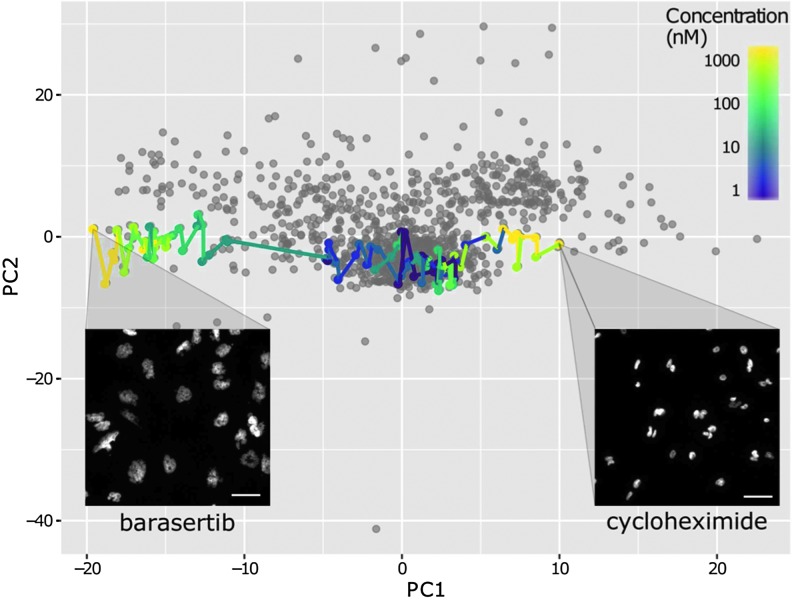
Phenotypic directions in the first two PCs. Scatter plot of the first two PCs of MDA-MB-231 cells treated with a small compound library. Principal component analysis was carried out on 309 median normalized features extracted from cellular images. Barasertib and Cycloheximide compounds are colored by concentration demonstrating opposite phenotypic directions in PC space producing opposite nuclear phenotypes. Images show nuclei imaged with Hoechst, scale bars: 20 μm. PC, principal component.

When the first two PCs are visualized as a 2D scatter plot, low concentrations of compounds are typically found near or within the DMSO cluster. However, with increasing concentrations, the points are often seen to proceed toward a given trajectory, describing decreasing phenotypic similarity to the negative control cells with increasing compound concentration. In the case of MDA-MB-231 cells treated with Cycloheximide and Barasertib, the compounds result in trajectories with opposing directions, describing opposite morphological changes (*Fig. 2*). The case of Barasertib and Cycloheximide provide a proof-of-principal example in the ability of the method described to distinguish opposing phenotypes represented by enlarged and aneuploidy nuclei characteristic of cytokinesis defects elicited by inhibitors of Aurora kinase B (Barasertib) in contrast to the condensed nuclei characteristic of the protein synthesis inhibitor (Cycloheximide).

**Fig. 3.**
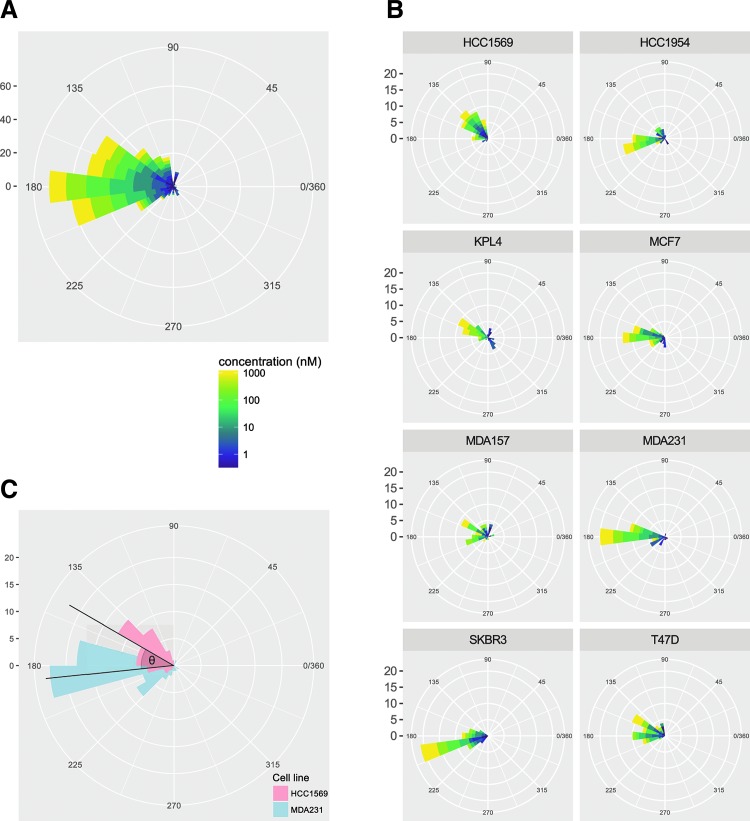
Circular histograms of theta values. **(A)** Circular histogram of theta values of Barasertib calculated for all eight cell lines. **(B)** Phenotypic direction of cell lines treated with Barasertib stratified by cell line. **(C)** A diagrammatic explanation of the theta value showing the difference in theta values between HCC1569 and MDA-MB-231 cell lines treated with Barasertib.

These distinct phenotypic trajectories have been quantified as theta values against a reference vector using Equation (1), where *u* is the PC1, PC2 vector, and *v* is the reference vector of (0, 1) (*Fig. 2*). A circular histogram of the theta values can then be plotted to visualize the distribution of compound induced phenotypes. The circular histogram theta plots provide an intuitive indication of a phenotypic direction produced by a specific pharmacological perturbation, as well as any change in phenotypic direction across increasing concentrations that may indicate off-target effects. *Figure 3A* shows a circular histogram of the data pooled from all eight cell lines treated with an eight-point half-log dose response of the Aurora B kinase inhibitor Barasertib. Using the same directional histograms, data can also be split by cell lines to directly visualize differential phenotypic response across a panel of distinct cell lines (*Fig. 3B*).

The difference in theta values between cell lines can then be calculated for a given compound to provide a univariate theta metric of phenotypic dissimilarity between cell types (*Fig. 3C*). It is possible to rank similarity and dissimilarity of each compound-induced phenotype between cells or between other compounds on a scale of 0–180° where 0 describes the most similar phenotypes and 180 the most dissimilar phenotypes. We name this method “Theta Comparative Cell Scoring” and provide the formula below:

\begin{align*}
\theta = \cos \left( { { \frac { u \cdot v }  { \vert \vert u \vert \vert v \vert \vert } } } \right) \times \frac { { 180 } }  { \pi } \tag { 1 } 
\end{align*}

### Screening for Differential Phenotypic Response Across the Panel of Breast Cancer Subtypes

To evaluate the TCCS method for the ability to identify compounds that induce differential phenotypic responses between the breast cancer cell lines, we calculated the difference between theta values for all eight breast cancer cell lines treated with 1 μM of 24 different compounds. Compounds were selected to represent 12 pairs of well-characterized mechanistic subclasses, 21 of these compounds elicited robust morphological changes in all eight cell lines.

**Fig. 4.**
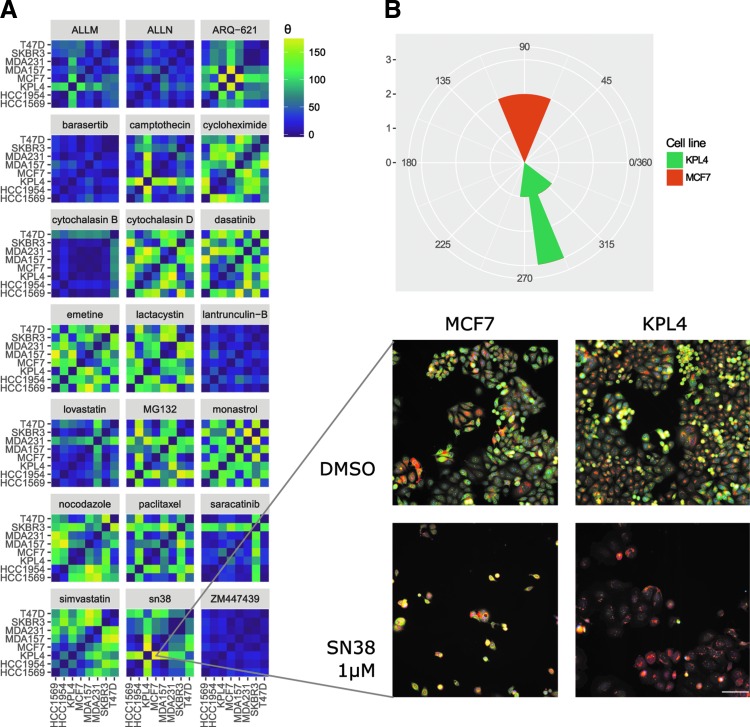
Heat map of theta values between pairs of cell lines for separate compounds. **(A)** Difference in theta values calculated between pairs of cell lines treated with 21 compounds at 1 μM concentration. Images show differential response between KPL4 and MCF7 cell lines treated with 1 μM SN38. MCF7 cells are observed to decrease in cell area, with bright staining for the endoplasmic reticulum, whereas the KPL4 cells produce a “fried egg” morphology with large spread cells and weak endoplasmic reticulum staining. Channels used are as follows: *Red*—MitoTracker DeepRed (mitochondria); *Green*—Concanavalin A (endoplasmic reticulum); *Blue*—Hoechst33342 (nuclei). Scale bar: 100 μm. **(B)** Circular histogram of theta values calculated for MCF7 and KPL4 cells treated with 1 μM SN38.

To identify and quantify differential phenotypic responses, the difference between theta values was calculated for all pairs of cell lines, constrained to the maximum dissimilarity value of 180° and plotted as a heat map for each of the 21 compounds (*Fig. 4*). Compounds with high theta values indicate a differential response between pairs of cell lines for that particular compound. A representative image between KPL4 and MCF7 cells treated with 1 μM of the topoisomerase I inhibitor SN38 is an example of a compound that induces a distinct phenotypic response between these cell types (TCCS = 179°), relative to the negative control for each cell line (*Fig. 4*). The majority of cell line comparisons returned low TCCS values, indicating that most of the breast cancer cell lines selected respond similarly to the compounds in our panel (*Supplementary Fig. S1*).

### Differential Response of Breast Cancer Cell Lines Are Stratified by Molecular Subclass

To demonstrate the ability of the TCCS method to cluster high-content phenotypic response across breast cancer subtypes with a view to informing disease positioning and personalized medicine strategies, we used data from an exemplar molecular targeted therapy, the dual Src/Abl inhibitor Saracatinib (AZD0530).

**Fig. 5.**
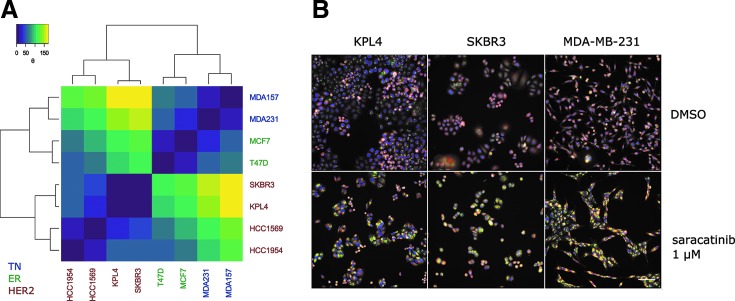
Heat map and hierarchical clustering of cell lines treated with Saracatinib. **(A)** Heatmap of TCCS values calculated between all pairs of cell lines treated with Saracatinib with hierarchical clustering by complete linkage of the Euclidean distance. **(B)** Images demonstrating two similar phenotypic responses—KPL4 and SKBR3—and the dissimilar phenotypic response of MDA-MB-231 cell lines to 1 μM Saracatinib treatment. Channels used: *Red*—MitoTracker DeepRed (mitochondria); *Green*—Concanavalin A (endoplasmic reticulum); *Blue*—Hoechst33342 (nuclei). Scale bar: 100 μm. TCCS, Theta Comparative Cell Scoring.

To utilize the data present across multiple titrations, the mean PCs were taken across eight concentrations to create the 2D vector with which the difference between TCCS values across all pairs of cell lines is calculated. TCCS values are plotted as a heat map clustered by hierarchical clustering using Euclidean distance (*Fig. 5A*). This revealed that the divergent high-content phenotypic response induced by Saracatinib across the breast cancer cell panel clustered together based on their molecular subclass. *Figure 5B* shows images of three cell lines treated with either DMSO negative control or 1 μM Saracatinib. From *Figure 5A* the MDA-MB-231 cell lines are found to have responded differently to KPL4 and SKBR3 cell lines, which in turn elicited a similar response to one another. This can be seen predominantly through increased cell–cell contact in the Saracatinib-treated MDA-MB-231 cells compared to the other two cell lines, observed as an increase in normalized number of adjacent cells in MDA-MB-231 cells (*Supplementary Fig. S2*). Although far from representative of all compound responses and disease subtypes, this example does indicate the potential of high-content cell-based phenotypic screening combined with application of the TCCS method across genetically defined cell panels to provide patient stratification hypothesis for both well-characterized candidate drugs or poorly characterized active compounds identified from phenotypic screens.

## Discussion

The rapid evolution and convergence of new technologies, including advances in image-based high-content phenotypic screening, induced pluripotent stem cell (iPSC) technologies, and gene editing, are well placed to advance a new era of modern phenotypic screening in more informative and disease relevant cell-based models of disease.^15,29,30^ However, a limitation of phenotypic screening is the identification of hit molecules or candidate drugs without knowledge of the target mechanism.

The lack of information on target mechanism, while not required for drug approval, impedes the design of personalized healthcare strategies to combat disease heterogeneity. Several target deconvolution strategies have been applied to compounds discovered by phenotypic screening to elucidate target mechanisms.^31–33^ However, no target deconvolution method is conclusive, and such strategies are often based upon the assumptions that a compound will only inhibit a single target and monitoring the activity and inhibition of the elucidated target will guide personalized therapy.

For the majority of compounds discovered by phenotypic screens, and for many complex human diseases where the one-drug-one-target hypothesis is unrealistic, new nontarget-centric approaches are required to understand drug mechanism-of-action and guide personalized healthcare strategies. *In vitro* pharmacogenomic or pharmacoproteomic profiling across well-characterized cell panels, representing specific disease subtypes, exemplifies one approach for informing drug mechanism-of-action and guiding personalized healthcare strategies in the absence of target knowledge. Breast cancer is separated into four major molecular subtypes; Luminal A (ER-positive and/or progesterone receptor (PR)-positive and HER2-negative and Low Ki67); Luminal B (ER-positive and/or PR-positive and HER2-positive or HER2-negative with high Ki67); Triple negative/basal like (ER- PR- and Her2-negative); and HER2 type (ER- PR- negative and Her2-positive). Each major molecular subtype of breast cancer can be further divided into subclasses based upon genetic mutation status and protein profiles, and the diagnosis of breast cancer subtype dictates the most appropriate personalized treatment for patients.^34–36^

In this article, we have developed a multiparametric high-content assay, data visualization tools, and a TCCS method, which support phenotypic screening of compound libraries across genetically distinct cells representing known molecular subtypes of disease. We provide proof-of-principle data applied to eight breast cancer cells representing four disease subclasses (*Table 1*), demonstrating the application of the method for quantifying distinct phenotypic response between cell types and clustering of cell-associated clinical subtypes based on similar or dissimilar phenotypic response to compound treatment.

As previously discussed, several multiparametric pathway and phenotypic profiling methods have been developed to classify drug mechanism-of-action and uncover new drug–target associations, and structure activity relationships in a more holistic and unbiased manner.^18,20–25,27^ However, the majority of these methods have been applied to single cell types amenable to high-content imaging or large-scale biochemical and proteomic analysis.^18,21–25,27^ The TCCS method described in this article was developed to provide a practical method to enable comparative multiparametric phenotypic analysis across a panel of genetically distinct cell types, which provides rapid quantification and visualization of divergent compound-induced phenotypic response between cell types. An intuitive explanation of the TCCS method would be the cosine distance in degrees of vectors in the first two PCs; this is a variation on existing methods that largely rely on correlation or Euclidean distance between compound vectors.^18^

The benefits of the TCCS over previous methods are as follows: (1) use of distance from the negative control to remove poorly active or inactive compounds that might produce spurious differences in correlation of cosine similarity measures; (2) The comparison of each data point to a common reference vector enables visualizations of a single metric, which depicts the relative change in phenotypic response induced by a compound (*Fig. 3A*).

The most critical aspect of comparing results between panels of distinct cell lines regardless of downstream methods is during the data preprocessing stage, which requires careful normalization against the negative control values for each cell line to remove inherent differences in cell line morphology. Thus, the TCCS method represents a flexible approach with broad applicability to quantifying and visualizing distinct phenotypes induced by a panel of compounds within a single cell type and/or the response of a single compound across multiple cell types. The TCCS method removes compounds from the algorithm that are not sufficiently different from the negative control. While this increases the robustness of the calculation, it also creates the opportunity to miss compounds that possess differential sensitivity between cell lines. This limitation of the method arises where certain compounds that do not induce any morphological change in one cell line may still perturb cellular morphology in another cell line, thus any such compound would subsequently be removed from the calculation due to insufficient distance from the negative control centroid, despite eliciting a genuine differential response between cell lines. However, this limitation can be simply rectified by implementing an initial preanalytical stage of the algorithm by calculating the distance from DMSO for all compounds across all cell lines to assign either as “active” or “inactive” phenotypic responders. Differences in the activation state of all compounds across all cell lines are recorded and the active compounds then progress to TCCS analysis to quantify and visualize a distinct phenotype response across cell lines.

The TCCS method as outlined in this article utilized only the first two PCs produced from the PCA. These two variables explain most of the variance of data in low dimensional data represented by majority of high-throughput high-content screens, which typically measure only small numbers of features.^37^ In such high-throughput compound screens, TCCS applied to the first two PCs would be expected to provide a single value describing the difference in response across different cell lines for active compounds. The method as applied to the first two PCs in this article becomes less informative in higher dimensional data sets as more PCs are required to describe the data. As the calculation to define the angle between two vectors [Eq. (1)] uses the dot product of the two vectors, the vectors are not limited to the first two PCs, and it is entirely reasonable that they could contain any number of PCs. Therefore, an alternative option would be to implement the TCCS method on a number of PCs that satisfy a user-defined proportion of variance within the data.

**Fig. 6.**
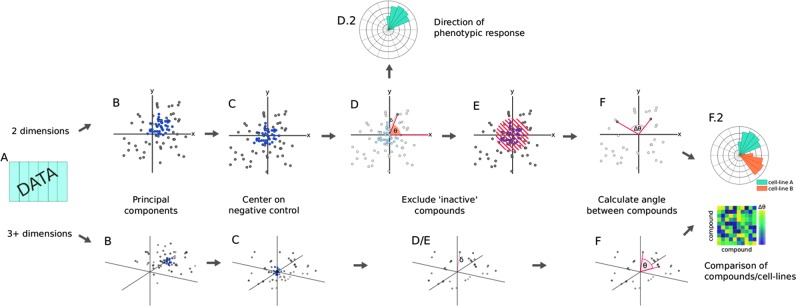
TCCS workflow. (A) Normalized numerical data. (B) PC analysis, negative control values colored in *blue*. (C) Centering of PC values to the negative control centroid. (D) Calculation of distance from the origin to each data point, an activity cutoff is derived from the standard deviation of the distance to the negative control values. (D.2) In two-dimensional space, a directional histogram can be created by the angle of each vector against a reference vector. (E) Inactive compounds excluded based on distance from the origin. (F) Determining the angle between compounds. (F.2) Visualization or clustering of compounds based on theta values.

Comparison of high dimensional vectors against one another rather than against a reference vector allows for direct calculation of a theta value in high dimensional space, an example workflow using the TCCS method applied to more than two PCs is provided in the online R scripts (github.com/swarchal/TCCS_paper) and is represented in the description of the TCCS workflow (*Fig. 6*). The TCCS method may also be applied to the normalized assay parameters rather than PCs as also demonstrated in the supplementary R workflow (github.com/swarchal/TCCS_paper). However, care should be taken to ensure that potentially uninformative parameters are not included in such analysis to avoid introduction of unnecessary assay noise. Thus, the most optimal application of the TCCS method can be appropriately tailored to each study and nature of the underlying high-content data set.

Multiple concentrations are not often used in high-throughput cell-based screening assays, despite providing useful information to detect off-target effects and can be thought of as inherent replicates of individual compound data. A further approach to incorporate titration data into defining direction in PC space would be to fit a linear model to each compound using simple linear regression, forcing the y-intersect through 0. While this would lose information pertaining to the distance from the DMSO centroid at each concentration, it would provide information regarding goodness of fit, and data may be excluded from the TCCS analysis if they do not fit the linear model well or used to indicate compounds with off-target effects at higher concentrations. As the theta value is essentially a direction in PC space, another useful addition would be to relate theta back to the feature loadings that describe how the PCs were constructed. This would return the phenotypic features that best describe a certain direction in phenotypic space. However, PCA contains negatively weighted features and so methods such as nonnegative matrix factorization in which the feature loadings are all positive values, may be a potential avenue for this improvement.

Another potential use of TCCS method is in assay quality control (QC). For example, TCCS could be applied to the simultaneous evaluation of two positive controls known to elicit robustly different morphologies (*e.g.*, paclitaxel and staurosporine) along with a negative control such as DMSO to determine a theta value between the two positive controls. It would be expected that the two positive controls would have a theta value greater than a specified minimum. The variance of theta values between two positive controls per plate could therefore be used as a measure of biological assay variability during assay development and screening campaigns.

Incorporating a multiparametric QC metric that utilizes high-content analysis across two positive controls provides increased robustness and more unbiased assessment of monitoring variation in cell behavior and assay variability over current methods that use a single positive control analysis of a preselected parameter. Other multivariate assay QC metrics typically build on the *Z*′-factor using supervised machine learning techniques such as Fisher's linear discriminant analysis (LDA) to best separate the positive and negative controls.^38^ Although more robust than single parametric analysis, a drawback of this method is that LDA is often prone to overfitting in high dimensions, which may produce overoptimistic QC values when processed to the *Z*′-factor calculation.

The convergence of new technologies, including next-generation sequencing, high-throughput proteomics, iPSC technology, and high-content phenotypic screening, is well placed to advance the identification of predictive biomarkers and personalized medicine approaches across a broader range of disease types and therapeutic classes.^15,29,30,39,40^

Our study provides a broadly applicable approach for quantifying distinct phenotypic response between genetically distinct cells using high-content analysis coupled to a TCCS scoring method. The TCCS method that we describe provides a univariate metric that can be applied to any high-content assay for quantifying and visualizing a diverse phenotypic response between cell types. The TCCS metric provides a univariate score of distinct phenotypic response on a scale of 0–180° (where 0° = similar and where 180° = most dissimilar), which can be used for correlation with orthogonal genetic, epigenetic, and proteomic datasets to support the identification of biomarkers of drug phenotype and further elucidate drug mechanism-of-action at genetic and pathway levels.

## Supplementary Material

Supplemental data

Supplemental data

Supplemental data

## Abbreviations Used

2D: two dimensional
3D: three dimensional
CCLE: Cancer Cell Line Encyclopaedia
DAPI: 4,6-diamidino-2-phenylindole
DMSO: dimethyl sulfoxide
EC_50_: concentration of drug that produces a 50% maximal response
ER: estrogen receptor
FITC: fluorescein isothiocyanate
GI_50_: concentration of a drug that gives half-maximal inhibition of cell proliferation
HER2: human epidermal growth factor receptor 2
iPSC: induced pluripotent stem cell
LDA: linear discriminant analysis
PBS: phosphate-buffered saline
PC: principal component
PCA: principal component analysis
PFA: paraformaldehyde
PI3K: phosphoinositide 3-kinase
PR: progesterone receptor
PTEN: phosphatase and tensin homolog
QC: quality control
TCCS: Theta Comparative Cell Scoring
TN: triple negative
WT: wild type
