# Supplementary material for: Development of the Theta Comparative Cell Scoring Method to Quantify Diverse Phenotypic Responses Between Distinct Cell Types
Source: Assay Drug Dev Technol. 2016 Sep 1;14(7):395–406. doi: 10.1089/adt.2016.730 (PMC5015429; doi:10.1089/adt.2016.730)
Supplement: Supplemental data [file Supp_Fig1.pdf]

SUPPLEMENTARY DATA

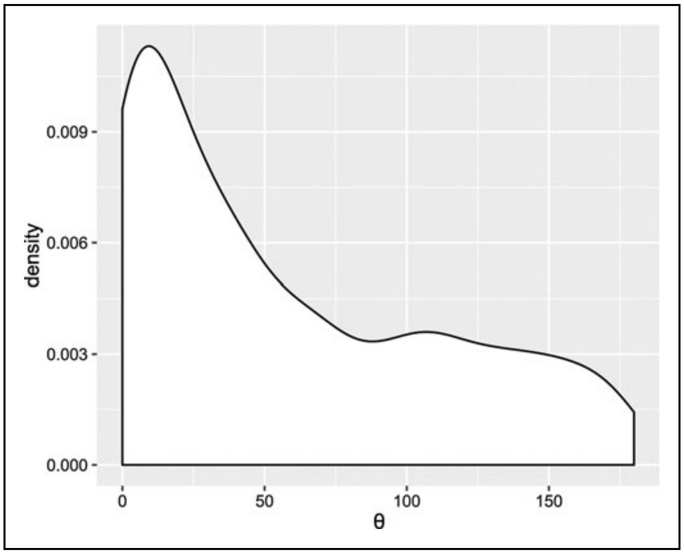

Supplementary Fig. S1. Density of theta differences between pairs of cell lines. A density plot of theta values from *Figure 4*, indicating most cell lines responded very similar to one another when treated with the same compounds, demonstrated by the abundance of low theta values.
