# Supplementary material for: Development of the Theta Comparative Cell Scoring Method to Quantify Diverse Phenotypic Responses Between Distinct Cell Types
Source: Assay Drug Dev Technol. 2016 Sep 1;14(7):395–406. doi: 10.1089/adt.2016.730 (PMC5015429; doi:10.1089/adt.2016.730)
Supplement: Supplemental data [file Supp_Fig2.pdf]

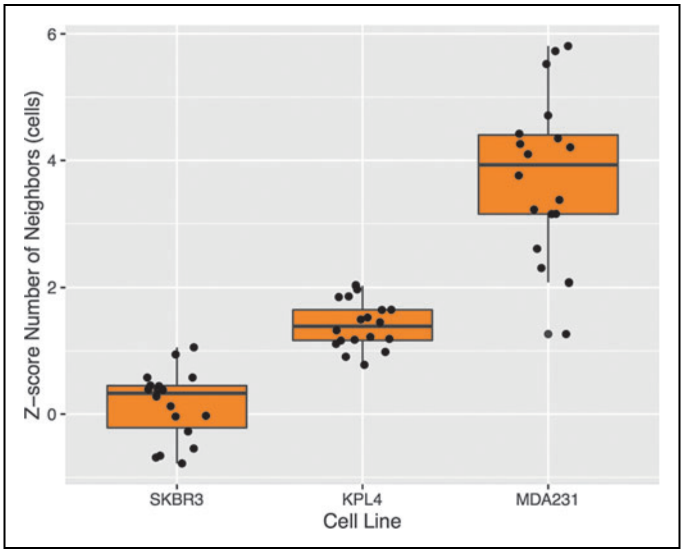

Supplementary Fig. S2. Box plot of number of cell neighbors. *Z*-scored values of number of cell neighbors for Saracatinib-treated cells, demonstrating the increased cell–cell contact in MDA-MB-231 cells compared to KPL4 and SKBR3 cell lines.
